# Permutation Entropy and Irreversibility in Gait Kinematic Time Series from Patients with Mild Cognitive Decline and Early Alzheimer’s Dementia

**DOI:** 10.3390/e21090868

**Published:** 2019-09-06

**Authors:** Juan-Andrés Martín-Gonzalo, Irene Pulido-Valdeolivas, Yu Wang, Ting Wang, Guadalupe Chiclana-Actis, Maria del Carmen Algarra-Lucas, Itziar Palmí-Cortés, Jorge Fernández Travieso, Maria Dolores Torrecillas-Narváez, Ambrosio A. Miralles-Martinez, Estrella Rausell, David Gómez-Andrés, Massimiliano Zanin

**Affiliations:** 1 Escuela de Fisioterapia de la ONCE, Universidad Autónoma de Madrid, 28034 Madrid, Spain; 2 Departamento de Anatomía, Histología y Neurociencia, Universidad Autónoma de Madrid, 28029 Madrid, Spain; 3 Visual Pathway Laboratory, Neuroimmunology Center and Neurology Department, Biomedical Research Center August Pi i Sunyer (IDIBAPS), Hospital Clínic Barcelona, 08036 Barcelona, Spain; 4 Unidad de Trastornos Cognitivos, Servicio de Neurología, Hospital Universitario Infanta Sofía, 28702 Madrid, Spain; 5 Paediatric Neurology, Vall d’Hebron University Hospital and VHIR (Euro-NMD, ERN-RND), 08035 Barcelona, Spain; 6 Centro de Tecnología Biomédica, Universidad Politécnica de Madrid, 28223 Madrid, Spain

**Keywords:** permutation entropy, irreversibility, gait, Alzheimer’s disease, mild cognitive impairment

## Abstract

Gait is a basic cognitive purposeful action that has been shown to be altered in late stages of neurodegenerative dementias. Nevertheless, alterations are less clear in mild forms of dementia, and the potential use of gait analysis as a biomarker of initial cognitive decline has hitherto mostly been neglected. Herein, we report the results of a study of gait kinematic time series for two groups of patients (mild cognitive impairment and mild Alzheimer’s disease) and a group of matched control subjects. Two metrics based on permutation patterns are considered, respectively measuring the complexity and irreversibility of the time series. Results indicate that kinematic disorganisation is present in early phases of cognitive impairment; in addition, they depict a rich scenario, in which some joint movements display an increased complexity and irreversibility, while others a marked decrease. Beyond their potential use as biomarkers, complexity and irreversibility metrics can open a new door to the understanding of the role of the nervous system in gait, as well as its adaptation and compensatory mechanisms.

## 1. Introduction

Within the large family of neurocognitive disorders, neurodegenerative dementias [1] are receiving increasing attention from the scientific community, to some extent proportional to their increasing prevalence in our ageing societies. "Neurodegenerative dementias" designates all conditions characterised by a progressive and slow deterioration of the central nervous system, causing symptoms related to cognitive decline (e.g., memory loss, language deterioration, impulsiveness, slow pace of thinking, difficulties in planning, orientation problems) as the main clinical feature. Dementia has devastating consequences both for the patients and their families, and globally for the whole society; to illustrate, the worldwide cost of dementia was estimated at United States (US) $818 billion in 2015 [2]. Neurodegenerative dementias have several etiologies, the most frequent of which is Alzheimer’s disease (AD), but which also include Parkinson’s, Huntington’s, prion, and motor neuron diseases.

According to the degree of disability, it is customary to distinguish between dementia (or major cognitive impairment) and mild cognitive impairment (MCI). MCI was forged as a concept to designate those patients with abnormal cognition, lying between normal ageing and very early dementia. Its importance is twofold. On one hand, MCI is a common condition, especially in developed countries, with high incidence (21.5–71.3 per 1000 people/year) and prevalence (3–42%) [3]. On the other hand, as most neurodegenerative dementias have a slow progression, MCI was also thought as an instrument for the early detection of patients at risk of future dementia. Numerous epidemiological studies have documented the accelerated rate of progression to dementia, and particularly to Alzheimer’s disease (AD), in MCI subjects [4,5]. Conversion rates range from 10–15% at specialised memory clinics and 6–10% in community-dwelling older adults [6].

In recent years, molecular, neurophysiological and imaging biomarkers have provided insights into the patient’s natural history, demonstrating a consistent profile during the full staging of AD (presymptomatic patients, MCI and dementia) [7,8]. Moreover, different indicators have shown potential in differentiating between MCI and dementia and/or in predicting the conversion of normal MCI into AD [9,10,11,12,13,14,15]. These findings have also been extended to other forms of dementia [16]. In spite of this, existing biomarkers are far from perfect and many shortcomings are recognised, these include, low diagnostic performance, the invasive nature of some tests, which are generally expensive or even unavailable in most healthcare facilities, low cost-effectiveness, and a global lack of understanding on how biomarkers from different sources should be combined in clinical practice.

A promising new approach is represented by gait analysis, as this is an altered domain in MCI and dementia, and as a consequence, its study may contribute to improving our understanding of the neurobiology of neurocognitive disorders. Alterations of gait occur in early phases of cognitive decline [17,18]. Recent studies support that changes in gait occur at the start of cognitive decline and may be detected in patients at risk of cognitive decline such as Apoε4 carriers [19]. Mean performance during mobility and gait-related tasks is strongly associated with cognitive decline, neurophysiological alterations [20,21] and brain volume changes in specific areas [22,23], and predicts the future risk of cognitive impairment in elderly subjects [24,25,26,27]. In comparison to healthy subjects, gait in MCI is featured by decreased velocity, shorter stride length, longer stride time, increased stride-to-stride variability [28,29,30] and spatiotemporal complexity [31].

Most of these reports stress the importance of spatiotemporal variables to distinguish MCI patients from healthy controls, but only few go deeper into the gait kinematics of patients with cognitive impairment [32]. Kinematics assesses the sequential configuration of the leg joints that are required to maintain the body’s centre of gravity above the stance base while moving forward. To avoid collapsing, multiple sequential configurations of the joints are dynamically changed through a gait cycle (i.e., the time between two heel strikes of the same foot) by muscle activation, which is controlled by neural mechanisms that depend on the integrity of somato-sensory, motor and cognitive integration cerebral networks. Gait is a cognitive purposeful action of high importance for the brain, and whenever those networks are damaged, the whole nervous system adapts its dynamics to achieve the target. Compensatory kinematic configurations then arise, in order to maintain walking capacity. Those newly-generated signals are a reflection of brain lesion/adaptation [33], and the quantitative evaluation of the differences between the MCI patients’, AD patients’ and the control subjects’ signals is an approach in which it is possible to study their neurobiology in more depth. These considerations, applicable both to dementia and other disorders, have led to the development of instrumental gait analysis (IGA), a set of techniques that are able to objectively quantify human gait, and whose use is gaining followers in clinical practice and research. IGA assesses patient’s specific problems by measuring how the body moves as a whole, by providing dozens of spatiotemporal parameters (e.g., walking speed or step length), and by further acquiring high-frequency kinematic measurements of those joints that align the lower extremity segments along the patient’s gait cycle [34]. Analysing those hundreds of parameters is not straightforward, and the solution may come from data mining techniques, which have enough power to classify and relate them to assess the effect of a condition [35,36].

As described above, some attention has been devoted to the study of changes in kinematic and spatiotemporal parameters obtained with IGA in conditions of cognitive impairment. However, no attempt has yet been made to analyse gait signals as time series, for instance to assess their complexity, which should be altered or modified as a result of motor system lesion and system re-adaptation in patients with cognitive impairment. The rationale behind this is that brain signals to the muscles which provoke joint movement may become less continuous and less coordinated; inefficient joint movement noise may be added as a consequence of failure of some neuronal networks; and those signals may produce poor configurations that may not respond (or may respond wrongly) to purposeful cognitive commands such as changing velocity.

Herein, we present a cross sectional study aiming at analysing the complexity of gait in a cohort of patients with cognitive impairment in the stages of MCI and mild AD (mAD). For this, we rely on two complementary metrics. On one hand, the complexity is measured through the well-known permutation entropy (PE) [37,38,39], a metric assessing the presence of causal relationships in the progression of a time series by looking at the underlying permutation patterns. On the other hand, we complement this information with a metric assessing the irreversibility of the time series, also based on the study of the appearance frequency of permutation patterns [40]. While PE and irreversibility have previously been used to study gait dynamics (see, for instance, [41,42,43,44,45,46] for PE, and [47] for irreversibility), to our knowledge this is the first time both metrics have been used to characterise gait kinematics in mild neurodegenerative dementias. The results depict a situation richer than that which was initially hypothesised. While these two metrics allow a distinction to be made between cognitive impaired patients and healthy subjects and impaired patients with different degrees of impairment, complexity and irreversibility are increased for some joint movements, but are reduced in others. We also show that PE and irreversibility yield partially complementary information, thus suggesting that they are measuring two different aspects of gait kinematics.

The remainder of the paper is organised as follows. Section 2 reports the main results obtained, including statistical and data mining-based analyses of the differences between the three considered groups of people. Afterwards, Section 3 presents a discussion based on these results, focused on the corresponding biomedical implications, and lays the ground for future works. Finally, Section 4 describes all the involved materials and methods, including a description of the patient cohorts (Section 4.1), of how data was acquired and pre-processed (Section 4.2), and how PE and irreversibility are calculated (respectively, Section 4.3 and Section 4.4).

## 2. Results

### 2.1. Complexity Measures are Related with Preferred Walking Speed and Cognitive Impairment

**Figure 1:**
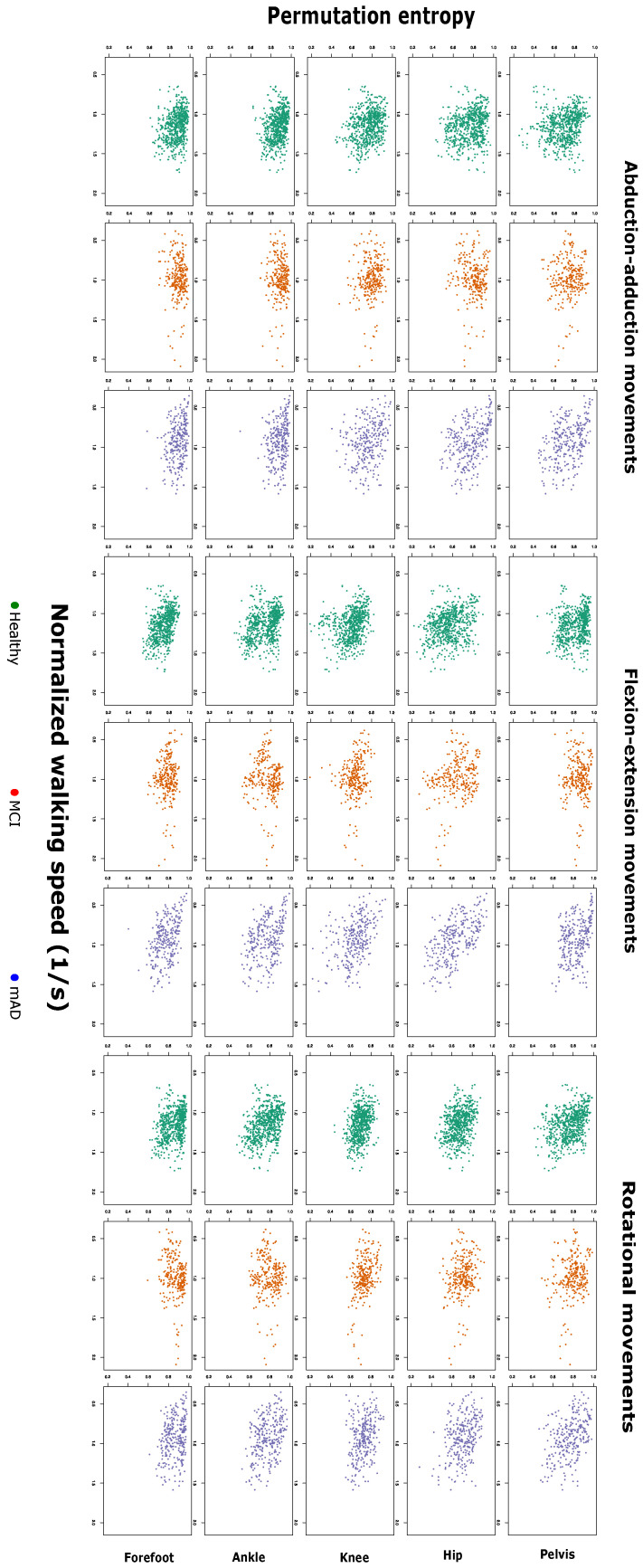
Single scale permutation entropy as a function of the normalised walking speed, for healthy subjects, mild cognitive impairment and mild Alzheimer’s dementia. Each panel corresponds to each joint and axis.

**Figure 2:**
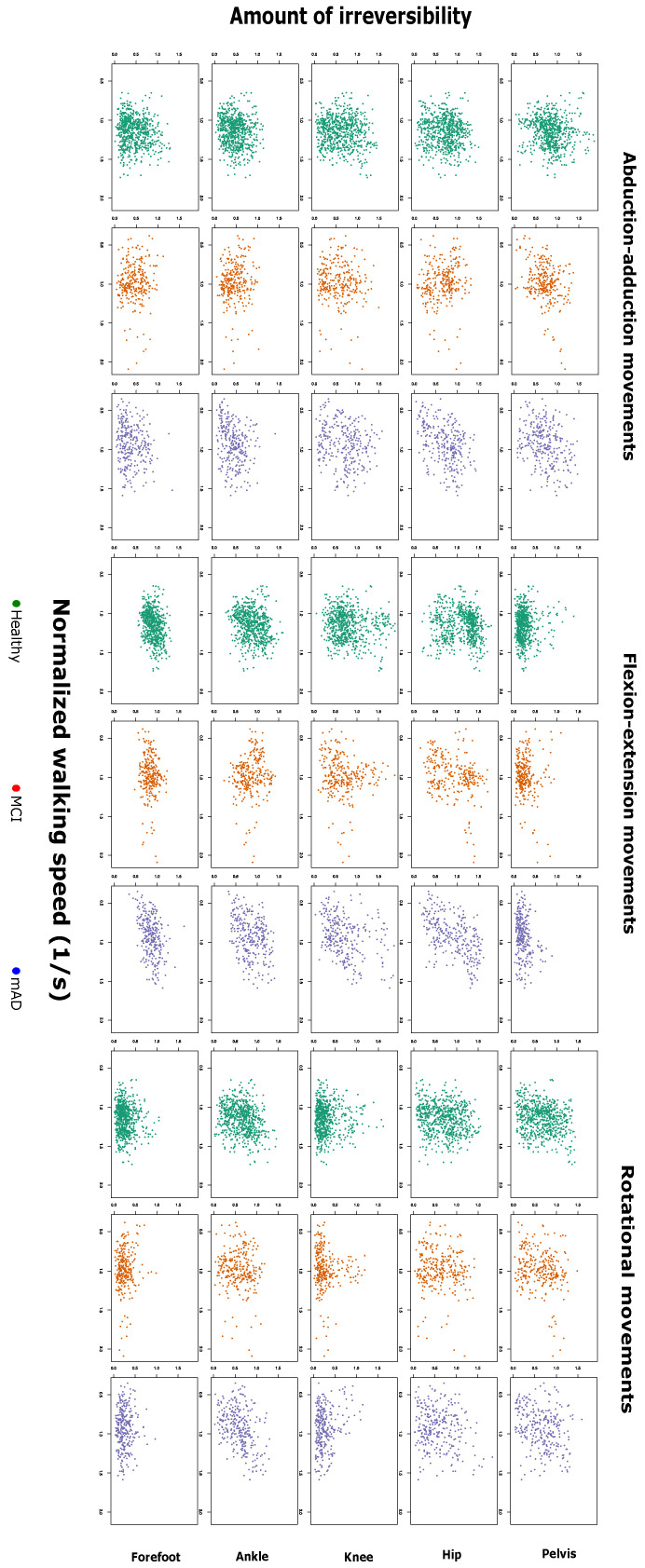
Irreversibility as a function of the normalised walking speed, for healthy subjects, mild cognitive impairment and mild Alzheimer’s dementia. Each panel corresponds to the same joint/axis as in Figure 1.

**Figure 3:**
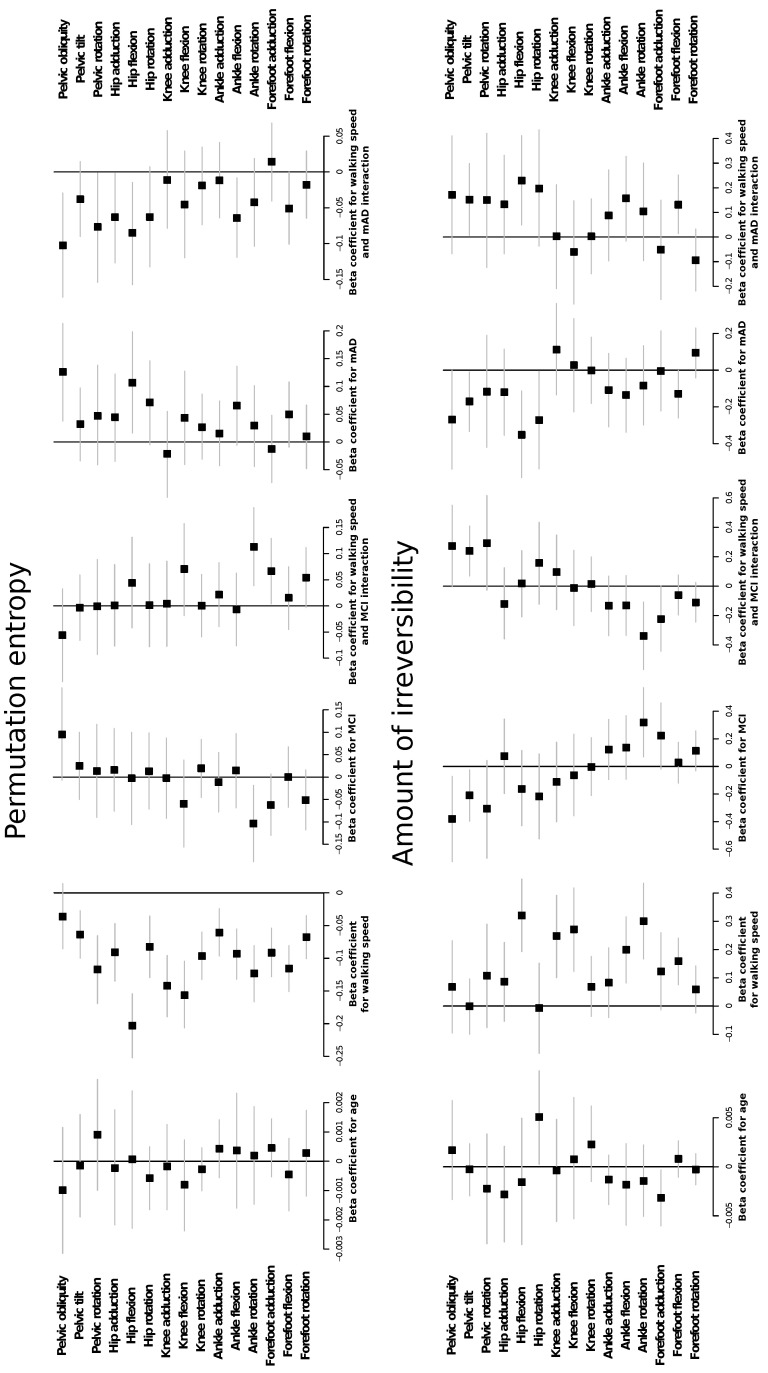
Forest plots showing the beta coefficients of linear mixed models comparing gait permutation entropy (PE) (upper part) and amount of irreversibility (IRR) (bottom part) values according to age, walking speed, disease status (mild cognitive impairment (MCI) vs. healthy and mild Alzheimer’s dementia (mAD) vs. healthy) and the interaction between walking speed and disease status. Squares represent the mean value of each beta coefficient, and horizontal lines the corresponding 95% bootstrap intervals.

Figure 1 and Figure 2 report the values of the permutation entropy (PE) and irreversibility (IRR) calculated on time series of healthy subjects, patients with mild cognitive impairment (MCI) and mild forms of Alzheimer’s dementia (mAD), according to the normalised walking speed. Figure 3 further shows the estimation and 95% confidence intervals of the beta coefficient from linear mixed models relating PE and IRR with age, preferred walking speed, group of cognitive impairment and the interaction between group of cognitive impairment and walking speed.

There is a limited effect of age on the PE and IRR of joint time series. Only IRR of hip rotation and forefoot adduction shows a significant effect, but with a relatively small effect size. The preferred walking speed influences both PE and IRR, but not in an equivalent way. PE of all joint kinematic time series, with the exception of the one referring to pelvic obliquity, shows a decrease for higher walking speed. In contrast, IRR is only increased at higher speed in hip flexion, knee adduction, knee flexion, ankle flexion, ankle rotation and forefoot flexion.

PE of the time series is generally not changed in the group of patients with MCI. Only PE of ankle rotation is decreased in patients with MCI, this effect being higher with decreasing walking speed. The effect of MCI on PE of forefoot adduction is dependent on the interaction with walking speed; specifically, at higher speeds, there is a decrease in the PE of forefoot adduction in MCI patients in comparison to healthy subjects.

PE of the kinematic time series shows different changes in the group of mAD. PE of pelvic obliquity and hip flexion shows a significant difference for mAD in comparison to healthy subjects, which is dependent on the walking speed. Specifically, patients with lower preferred walking speed show a higher increase in PE of these two time series. Moreover, the decrease in PE of ankle flexion at higher walking speeds is more pronounced in patients with mAD with respect to healthy subjects.

IRR of pelvic tilt shows different changes in the MCI and mAD groups. There is a decrease with respect to healthy subjects if walking speed is not considered, but IRR rises higher than in healthy subjects (for whom there was no effect of WS) when WS increases. In the case of MCI, IRR of ankle rotation is increased in comparison to healthy subjects, but this difference decreases at higher walking speeds. In the case of mAD, there are significant effects for IRR of hip flexion, hip rotation and ankle flexion, which are lower than in healthy people, this difference being less marked as WS increases (with the exception of hip rotation, for which the beta coefficient of the interaction is not statistically different from zero).

### 2.2. Random Forests Detect a Distinguishable Pattern between the Different Groups of Cognitive Impairment

**Figure 4:**
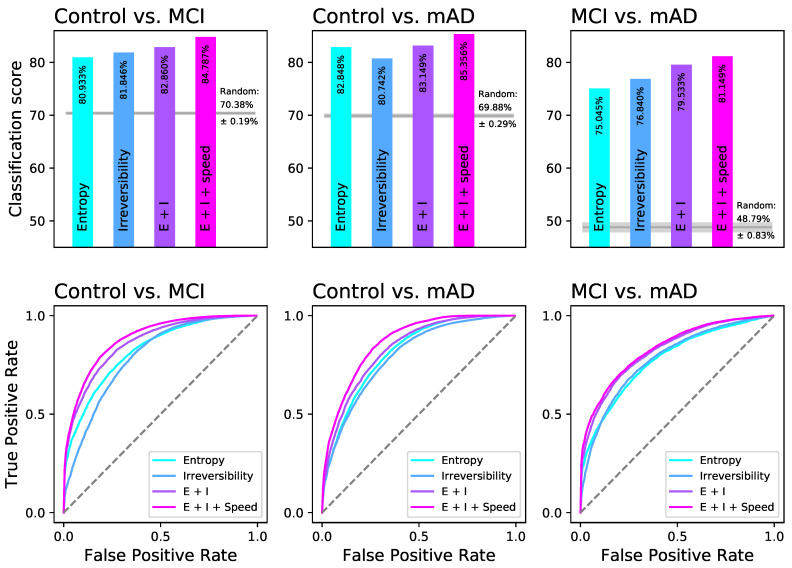
Results of the classification tasks. (Top) Classification score obtained by random forests based on permutation entropy, irreversibility, combination of permutation entropy and irreversibility (*E + I*), and combination of entropy, irreversibility and preferred walking speed (*E + I + speed*). The grey horizontal lines report the results of a classification in which data are randomly shuffled. (Bottom) Average ROCcurves, grouped according to the three considered tasks. See main text for details.

In contrast to linear mixed models, random forests are able to capture multidimensional patterns, which may help to detect more complex differences in entropy and irreversibility for different degrees of cognitive impairment. Herein, we report the performance of random forests trained with real data, in terms of the classification score, and compare it with the performance of random forests trained with 10 randomly shuffled data sets—see Figure 4, top panels.

Using either entropy or irreversibility of kinematic time series, random forests detect differences between groups, with a classification score between 75% and 83%. The prediction capacity is higher for separating healthy subjects from cognitive impaired groups than for discriminating between MCI and mAD patients. The combination of entropy and irreversibility improves the classification performance, particularly if the preferred walking speed is included in the algorithm. These prediction scores are around 80–85%, which are good enough to support that there are differences in the patterns of complexity between groups, although an overlap is still present.

The bottom panels in Figure 4 further report the classification score in the form of ROC curves. In order to obtain representative results, the classification was performed using half of the instances (randomly drawn) for training and half for the testing; the process was repeated 100 times, and the resulting ROC curves averaged. It can be appreciated that the classification with both entropy and irreversibility always yields higher curves than those only considering one single metric, thus confirming the previously shown results.

### 2.3. Permutation Entropy and Irreversibility Yield Complementary Information

**Figure 5:**
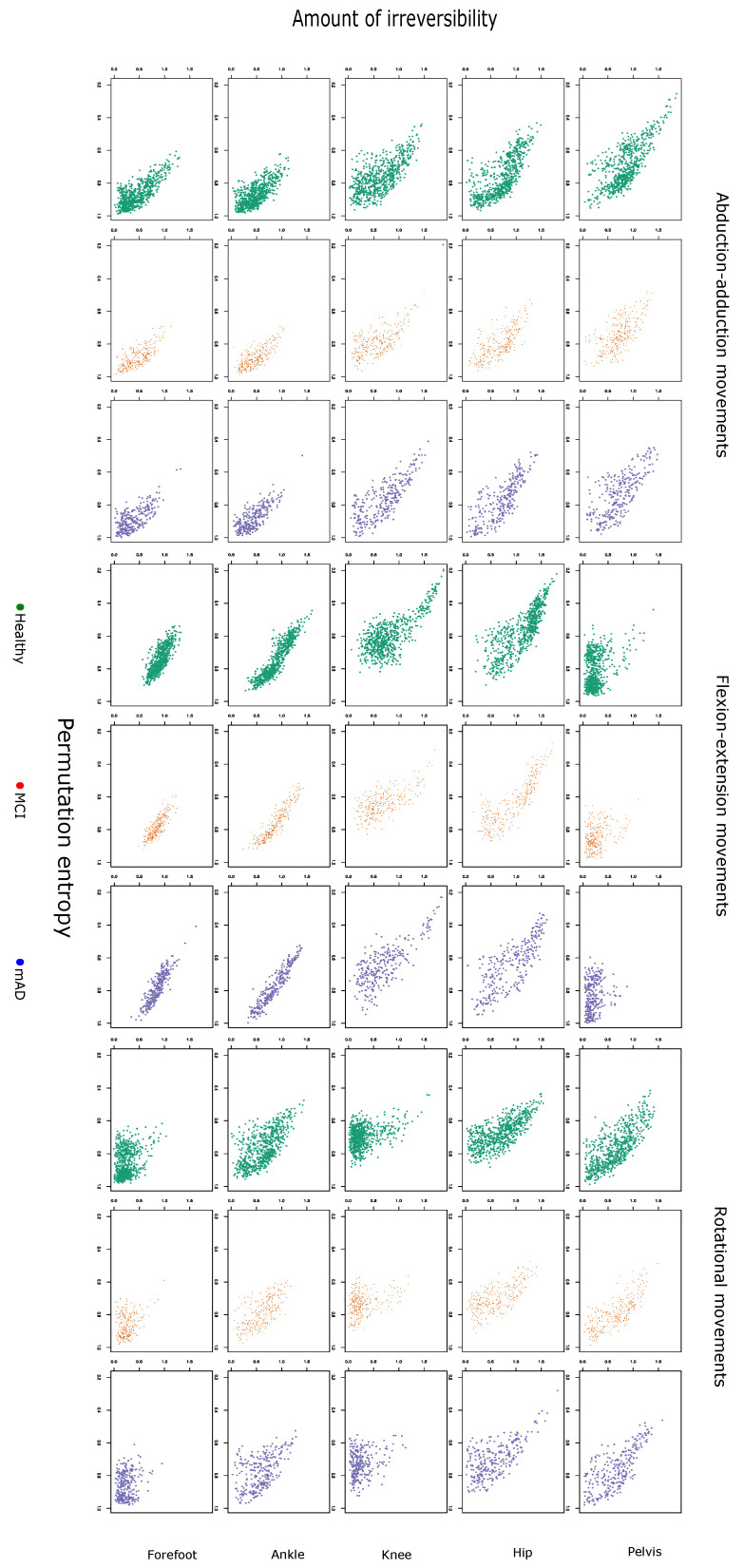
Irreversibility as a function of the permutation entropy, for healthy subjects, mild cognitive impairment and mild Alzheimer’s dementia. Each panel corresponds to the same joint/axis as in Figure 1.

**Figure 6:**
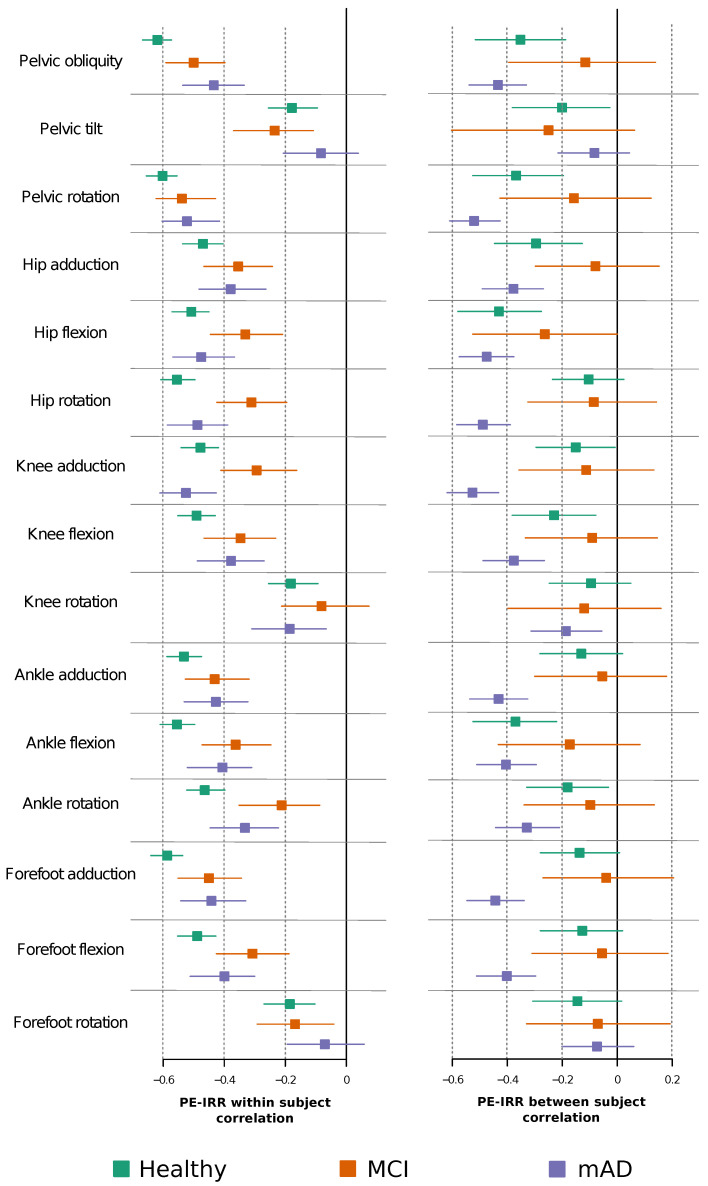
Forest plots showing the correlation coefficient between permutation entropy and the amount of irreversibility, controlled by age and walking speed. Left and right panels respectively report within-subject and between-subject correlations. Squares represent the mean value of each beta coefficient, and horizontal dashed lines the corresponding 95% bootstrap intervals. Different colours are used to show the different groups.

In Figure 5, we show a scatterplot of PE and IRR for the time series of each joint movement for the same gait cycle. To analyse the correlation between PE and IRR, we calculated two coefficients: one corresponding to the within-subject variance, and a second one to the between-subject variance. Figure 6 shows that, in general, within-subject correlations are significantly higher than between-subject correlations. Moreover, most within-subject correlations show statistical significance; in contrast, in the case of between-subject correlations, we found statistical significant coefficients only for some kinematic joint time series. It is worth noting the variable degree of correlation, mainly depending on the type of joint kinematic time series. In the case of the within-subject correlation, there are small differences between groups with different degrees of cognitive impairment. On the other hand, between-subject correlation coefficients are more sensitive to the group of cognitive impairment.

## 3. Discussion

In this paper, we have shown that PE and IRR of joint kinematic time series along the gait cycle are modified in two stages of cognitive decline. Both PE and IRR depend on the walking speed. Moreover, the differences throughout different levels of cognitive impairments are also dependent on preferred walking speed, which is decreased in some patients. Interestingly, the effect of ageing seems more limited than the impact of cognitive dysfunction and walking speed. We have also demonstrated that the differences in kinematic complexity may allow discrimination between groups with different cognitive involvement. Lastly, we have demonstrated that PE and IRR provide complementary properties to provide more in depth insight into the complexity of gait kinematics. The relationship between PE and IRR in a gait cycle depends on the joint described by the kinematic time series, and on the group of patients in which it is studied.

In biological terms, measures of complexity such as PE and IRR may help in better understanding the processes of motor impairment/adaptation that occur in people with cognitive impairment. There are changes in complexity that are shared by both groups of patients: for instance, the amount of irreversibility of the pelvic tilt kinematic signal is decreased in both groups (MCI and mAD), being lower in patients with lower walking speed, thus denoting a decreased complexity of this joint movement in patients with slower gait. Interestingly, there are changes in complexity that are not shared by both groups of patients. We have shown that there is an increase in complexity at distal joints in MCI (decreased PE of ankle flexion and increased IRR of ankle rotation) that is higher in patients with MCI and lower speeds, and that is yet practically absent in patients with mAD. In parallel, patients with mAD show a decreased complexity for proximal joints (increased PE in the hip flexion and pelvic obliquity kinematic signals and decreased IRR of hip flexion and hip rotation) and for the forefoot flexion. These differences are again sharper in patients with lower WS. The increase in differences in patients with lower WS deserves special consideration. Two hypotheses can be proposed. Firstly, patients with cognitive impairment may use different kinematic complexity when they walk more slowly. Secondly, patients who walk more slowly may also be more severely affected in terms of motor capacity, with a consequent stronger alteration of kinematic complexity. It is difficult to check which one of these two hypotheses is true with our experimental design; further studies are needed to clarify how patients with cognitive decline adapt their gait complexity to different walking speeds.

The discrimination between groups based on complexity measures of kinematic joint time series is also a finding of biological significance. It is worth highlighting that, while it is in theory possible to build a diagnostic tool based on the presented results, this is not the aim of this work. On the contrary, classification models are here used to quantify the differences in gait adaptations in people with mild cognitive alterations. The presence of significant motor alterations in early/mild phases of cognitive deterioration, and the understanding of their biological foundations, is relevant for interpreting changes in later phases of cognitive impairment. Previous studies demonstrated that motor impairment slowly progresses from early phases, although symptoms were believed to only arise in later phases [17]; in contrast, our results support that kinematic disorganisation is also present at early phases of cognitive impairment. This makes a case for the use of complexity measures in future studies and in the creation of gait-based biomarkers, possibly in conjunction with simpler acquisition technologies, e.g., wearable accelerometers.

Beyond widening the methodological framework to study complexity of gait kinematic time series, the paper’s main contributions are that (*i*) gait complexity changes at early mild cognitive impairment, and (ii) changes in gait complexity differ between MCI and mAD.

From a purely methodological point of view, our study also demonstrates that PE and IRR provide information about complementary properties in the evaluation of biological time series. By separately analysing within- and between-subject correlations, we have shown that PE and IRR share a higher proportion of intra-subject variance, which means that changes within the same subject in PE are also accompanied by changes in IRR. The correlation is far from being perfect, and we have shown that it varies depending on the considered joint time series. In contrast, between-subject correlation is generally low, which means that, excluding the potential effect of preferred walking speed, a subject with higher PE will not necessarily have a lower IRR. This implies that IRR and PE are reflecting different system and gait processes.

Some limitations of the proposed work have to be acknowledged. Mild cognitive decline and mild Alzheimer’s dementia were defined according to clinical criteria, i.e., without the use of biomarkers and advanced imaging protocols. While this represents the current clinical practice in most health centres, this limits the possibility of correlating gait changes with this kind of biomarkers. As a second limitation, our study has a transversal design, while some features of cognitive decline have a dynamic behaviour. Even though this does not detract from the contributions of our study, relationships between complexity and future progression of the patients can only be hypothesised. Finally, it is worth noting that this study is non-interventional, as patients are observed while using their preferred walking speed. We did not explore whether complexity measures of gait kinematics could change due to external interventions or to modified walking speed; this prevents the interpretation of the observed relationships as causal ones. Such an interventional approach can be an interesting paradigm for future studies, relating changes in complexity with adaptations to different gait spatiotemporal outputs.

In conclusion, our study uses PE and IRR to characterise the complexity of gait kinematic time series in mild cognitive decline and mild Alzheimer’s dementia. We found that there is a pattern of complexity measures that distinguish cognitive impaired patients from healthy subjects and impaired patients with different degree of impairment. The pattern is characterised by increased complexity in some joint movements and decreased complexity in others. We also show that PE and IR may be partially complementary indices, capturing different aspects of complexity in gait kinematics time series.

## 4. Materials and Methods

### 4.1. Participants

**Figure 7:**
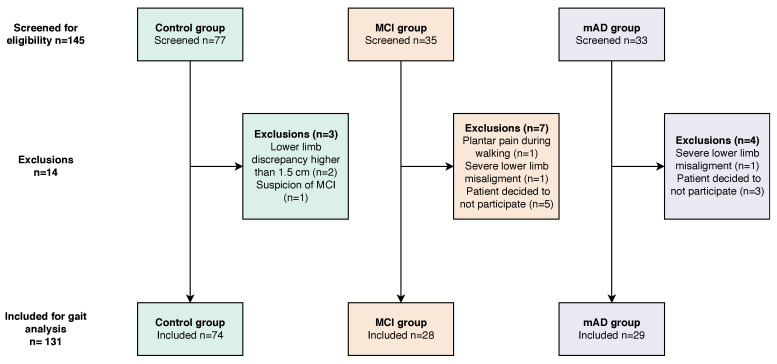
Flow diagram indicating the screening and selection of participants.

The present study relies on kinematic data recorded for three groups of people, i.e., MCI, mAD and matched control subjects. Figure 7 depicts a flow diagram indicating the recruitment and selection process of subjects; additionally, Table 1 shows the features of the finally included subjects.

We recruited two groups of patients with cognitive impairment, one with MCI and a second one with mAD. Thirty-five MCI patients and 33 patients with mAD were screened, but finally 28 and 29 were finally included. MCI and mAD patients were recruited in the memory unit of Hospital Universitario Infanta Sofía, Madrid, Spain, and had been diagnosed according to Petersen’s criteria [4] and 2011 NIA–AA diagnostic criteria of probable AD [48], respectively. Patients were evaluated following a protocol that included a personal interview, medical history, full neurological examination, brain imaging, assessment of the global impact of the cognitive impairment by means of clinical dementia rating [49], evaluation of behavioural problems by means of the Blessed dementia rating scale [50], brain imaging and a battery of neuropsychological tests, including at least the MMSE test [51,52], digit span test [53,54], digit inverse test [53,54], free and cued selective reminding test [55,56], clock drawing test [57] and Rey–Osterrieth complex figure (copy and memory) [56,58]. Inclusion criteria for patients were:

- Age lower than 75 years;
- Absence of a diagnosis of moderate or severe dementia;
- Absence of clinical suspicion of rapidly progressive dementia;
- Absence of previous stroke within six months or previous stroke without full recovery;
- Absence of an active and non-related diagnosis of a psychiatric or neurological disorder that may impair gait;
- No suspicion of rapidly progressive dementia;
- Not having history of previous stroke within six months or focal findings attributed to a previous stroke;
- No previous psychiatric or other neurological disorders that may impair clinical evaluation or gait analysis;
- Absence of a current diagnosis of an inter-current systemic neurological or cardio- respiratory disease;
- Absence of severe visual or auditory disability;
- Absence of surgical treatment in lower limbs within the previous year;
- Ability to walk seven meters without external support;
- Satisfactory family environment.

Ninety matching voluntary subjects were recruited ad hoc to build a control group with no cognitive complaints and no abnormalities in the mini mental state examination (MMSE) [51]. Inclusion criteria included:

- Age between 50 years and 75 years;
- Absence of orthopaedic lesions or major surgery within the previous five years;
- Absence of cognitive complaints;
- Absence of a current diagnosis of an inter-current systemic neurologic or cardio- respiratory disease;
- Absence of severe visual or auditory disability, and
- A score higher than 28 points in the MMSE test [51,52].

Control subjects had a similar age and sex distribution to the groups with cognitive impairment. Two subjects were discarded for orthopaedic or clinical reasons, making a final number of 87 volunteers.

Note that the two groups of patients do not match in number due to the limited availability of participants that fulfilled the inclusion criteria. However, the size of both cohorts is large enough to support the statistical significance of our results. Additionally, group imbalance has been taken into account in the classification tasks, through the execution of the same tasks on randomly shuffled data.

Our local Ethics Committee approved this study and individuals were all subjected to examination after informed written consent. The work was carried out in accordance with the Code of Ethics of the World Medical Association (Declaration of Helsinki).

### 4.2. 3D Gait Analysis and Data Preprocessing

Gait analysis was performed with a Codamotion system (Charnwood Dynamics Ltd., Rothley, UK). Twenty-four light emission markers were attached to the same number of positions of the subject’s legs, according to an anthropological segment model designed by the manufacturer, and signals were recorded at 200 Hz while the subjects were performing the task. Subjects were incited to walk 10–15 times from one end to the other of a 7 m long walkway path (between 5 and 7 gait cycles per walkway) at their natural, spontaneous speed. The system acquired continuous real-time kinematic data during each complete walk over the walkway. After the acquisition session, individual gait cycles were isolated, by manually marking their beginning (heel contact) and their end (next heel contact of the same foot). Cycles were then reviewed to select those in which the gait was more stable, which usually coincided with those obtained from the 3–5 central meters of the walkway. Next, each selected cycle was again reviewed to check the consistency of the signal reception. The whole post-acquisition selection process was performed by two independent reviewers, with the help of a custom software programmed in R. This program is designed to detect abnormalities in cycle marking or signal reception, and to eliminate outliers in discrete kinematic parameters that might mean marker failure or displacement, and which might have escaped the manual revisions. This data validation process resulted in 25 valid cycles from each side (left or right leg of the individuals). For every side gait cycle, we studied the time series (201 time epochs) of the 3 angular planes (sagital, horizontal and coronal planes) from 5 joints (pelvis, hip, knee, ankle and forefoot).

### 4.3. Permutation Patterns and Entropy

The concept of permutation patterns, and especially of permutation entropy (PE), was introduced by Bandt and Pompe in a seminal paper in 2002 [37]. In short, given a time series, PE is based on the idea of finding the order patterns that result in sorted (ascending) sub-sequences, and of then studying the probability distribution (and eventually the entropy) of these patterns. PE then allows assessment of the temporal causality of a time series, in a computationally efficient and almost parameter-free way. Since its introduction, PE has been used to tackle multiple problems, from estimating the complexity of a time series, to identifying the nature (chaotic vs. stochastic) of the process generating it. While a brief mathematical formulation is provided below, the interested reader can find further information in several reviews, e.g., [38,39].

Let us start with a time series $X=\{x_{t}:t=1\ldots N\}$, thus composed of *N* data points. A vector of consecutive points can be associated to each time index *t*, such that $s_{t}\mapsto \left(x_{t},x_{t+1},\ldots ,x_{t+D+1}\right)$. Note that we here fix *D*, also known as the *embedding dimension*, to 3, due to the limited length of the available time series [59]. We additionally consider perfectly overlapping time series—i.e., we fix τ=1, according to the standard PE formalism [38].

Each vector can then be associated to a *permutation pattern*, defined as the permutation that should be applied to the vector to obtain a sorted sequence. To illustrate, suppose that X={0,3,2,5,1,…}; $s_{1}$ will then be defined as $s_{1}=\left(0,3,2\right)$, and the corresponding permutation pattern will be π(132)—as the smallest element is the first, followed by the third and the second, i.e., $x_{1}<x_{3}<x_{2}$. It is easy to see that only D! patterns can appear—and, in this case, 3!=6. One can then construct a probability distribution $p(\pi_{1},\ldots ,\pi_{6})$, and define the normalised permutation entropy through the Shannon entropy of *p*:

(1) $$PE=-\frac{1}{\log_{2}D!}\sum_{i=1}^{D!}\pi_{i}\log_{2}\pi_{i}.$$

As has exhaustively been studied, PE values close to 1 suggest the presence of stochastic (random) processes; on the other hand, 0≤PE<1 implies the presence of some temporal causality in the generating dynamics, such as, for instance, a chaotic behaviour.

### 4.4. Irreversibility of Time Series

In mathematical terms, a time series can be defined as *irreversible* whenever there is a lack of invariance of its statistical properties under the operation of time reversal [60,61]. Thus, given a time series $X=\{x_{t}:t=1\ldots N\}$, its time reversed version $X^{t.r.}=\left(x_{N},\ldots ,x_{1}\right)$, and a generic function *f*, X is said to be irreversible if $f\left(X\right)\neq f\left(X^{t.r.}\right)$. More intuitively, an irreversible time series is one in which the observer can easily identify a time arrow, or a preferred temporal direction in the progression [61]—as is the case of a movie showing a glass falling and breaking on the ground. Irreversibility can be due to different causes, as for instance nonlinear dynamics, non-Gaussian (linear or nonlinear) generative models, or in general the presence of a memory [62]. Assessing irreversibility is therefore tantamount to assessing the degree of time series predictability and non-linearity; it is thus conceptually not distant from the idea underpinning PE.

We here measure the irreversibility of gait dynamics through the metric based on permutation patterns recently proposed in [40]. Note that several similar methods have been proposed in parallel, as for instance in [63,64]. Starting from a time series X and the corresponding permutation patterns $\pi_{t}$, it is based on the idea that each pattern maps to a different one under the operation of time reversal. For instance, if at time *t* the pattern π(123) is found, reversing the time series will necessarily imply that $\pi_{t+2}=\left(321\right)$—note that a constant of 2 is added to account for the embedding dimension, here fixed to D=3. To illustrate, if s=(4,5,6), and hence π=(123), its time reversal will necessarily be $s^{t.r.}=\left(6,5,4\right)$, thus leading to $\pi^{t.r.}=\left(321\right)$. A simple test can then be designed: the time series is irreversible when $p(\pi_{123})$ is different from $p(\pi_{321})$ in a statistically significant way, as the relative abundance of any of those two patterns can be used to define a preferred time direction. Note that a similar argument holds for the pairs of patterns ($\pi_{213}$, $\pi_{312}$) and ($\pi_{231}$, $\pi_{132}$).

Using a similar idea, one can easily calculate the *amount of irreversibility* of a time series. Specifically, let us consider the distributions $P_{f}=\left(\pi_{123},\pi_{213},\pi_{231}\right)$ and $P_{b}=\left(\pi_{321},\pi_{312},\pi_{132}\right)$, the latter being equivalent to the former under the operation of time reversal. The more $P_{f}$ and $P_{b}$ differ, the more clear is the irreversibility of the time series. This can easily be quantified through the Kullback–Leibler divergence:

(2) $$D_{KL}=\sum_{i=1}^{3}P_{f}\left(i\right)\log\frac{P_{f}\left(i\right)}{P_{b}\left(i\right)}.$$

Values of $D_{KL}$ close to zero indicate that the time series is reversible; on the other hand, the higher is $D_{KL}$, the more irreversible the time series is.

### 4.5. Effect of Cognitive Decline on Permutation Entropy and Irreversibility of Every Joint Kinematic Time Series: Univariate Study

We estimated the mean difference in the permutation entropy and the amount of irreversibility of the kinematic joint time series between groups (using healthy subjects as reference) by means of mixed effect models. We considered subjects and side nested on subject as random intercept. As fixed effects, we included group according to cognitive performance, age, normalised walking speed and the interaction of normalised walking speed and the cognitive group. This analysis was performed using package *lmer* in R. We used beta coefficients for fixed effects as effect statistics, and their 95% CI were calculated by means of parametric bootstrapping.

### 4.6. Correlation of Permutation Entropy and Irreversibility of Every Joint Kinematic Time Series in Each Joint Time Series

We used Bayesian, bi-response regressions for continuous variables programmed with non-informative priors (2,300,000 iterations of the Markov chain Monte Carlo algorithm with a burn-in of 300,000, and thinning of 100) using the *MCMCglmm* package in R [65] to calculate the degree of relationship between permutation entropy and irreversibility in every joint kinematic time series from each group, and, in that way, assess how much independent information permutation entropy and amount of irreversibility are providing. On the basis of variance–covariance matrices of the models, we calculated two correlation coefficients: one within-subjects and one between-subjects using the method described by Dingemanse and Dochtermann [66]. We used the 95% highest posterior interval as credible interval.

### 4.7. Classification Tasks

Classification tasks were performed through the well-known random forest (RF) model [67]. This model is constructed by merging an ensemble of decision tree classifiers, each one trained on a different and random subset of features and instances; the final classification is made by choosing the output class selected by the majority of the trees. The choice of the RF model was guided by three advantages [68]: (*i*) its recognised accuracy in classification tasks, usually superior to other models; (*ii*) its resilience to overfitting, especially in the case of limited availability of instances in the training phase; and (*iii*) its computational efficiency. In all tasks, we used the implementation included in the *scikit-learn* Python library [69]. Except for the number of trees, fixed to 1.000, all other parameters have been left at the default value.

In order to estimate the generalisation accuracy of all models, a leave-one-out cross-validation (LOO CV) strategy was implemented [35]. An independent model was trained *N* times, with *N* being the number of instances in the data set. All data, except for those corresponding to a single instance, were used for training, for then for obtaining the prediction on the excluded instance. The final accuracy was then calculated as the fraction of times (i.e., of models) corresponding to a correct classification.

## Figures and Tables

**Table 1:** Features of subjects involved in the study. Note the differences between MMSE score, normalized walking speed and stance time. Abbreviations: MMSE: mini-mental state examination, Q1: Quartile 1 Q3: Quartile 3. * Osteoarthritis was asymptomatic in the moment of the gait analysis without pain and passive limitation at physical examination.

	Healthy Subjects (n = 74)	Mild Cognitive Decline (n = 28)	Mild Alzheimer’s Dementia (n = 29)	*p*-Value
**Age** (years) [median (Q1–Q3)]	63.2 (11.2)	69.1 (5.2)	67.8 (5.49)	0.15
**Female** [n (%)]	42 (53%)	16 (57%)	17 (59%)	1
**Body mass index** (kg/m${}^{2}$) [median (Q1–Q3)]	26.91 (24.13–30.83)	27.72 (23.13–31,25)	26.04 (24.02–28.3)	0.247
**MMSE** [median (Q1–Q3)]	30 (29–30)	25.5 (22–27)	20 (18–23)	<0.001
**Education level** [n (%)]				0.861
*No studies*	3 (4.1%)	1 (3.6%)	2 (6.9%)	
*Basic studies*	51 (68.9%)	23 (82.1%)	22 (75.9%)	
*Intermediate studies*	8 (10.8%)	2 (7.1%)	2 (6.9%)	
*University studies*	12 (16.2%)	2 (7.1%)	3 (10.3%)	
**Time with cognitive complaints** [median (IQR)]	-	12 (6–24.25)	13 (7–24)	0.636
Knee osteoarthritis * [n (%)]	0 (0%)	0 (0%)	1 (3.4%)	0.435
Hip osteoarthritis * [n (%)]	0 (0%)	0 (0%)	1 (3.4%)	0.435
**Normalised walking speed** (s${}^{-1}$) [median (Q1–Q3)]	1.13 (1.03–1.28)	0.99 (0.86)	0.94 (0.77–1.09)	<0.001
**Cadence** (steps/s) [median (Q1–Q3)]	1.63 (1.53–1.75)	1.5 (1.43–1.66)	1.5 (1.39–1.59)	0.352
**Stance time** (% gait cycle) [median (Q1–Q3)]	65 (64.1–66)	67.1 (65.7–69.1)	66.9 (66–70.4)	<0.001

## Abbreviations

The following abbreviations are used in this manuscript:

AD	Alzheimer’s dementia
IGA	Instrumented gait analysis
IRR	Amount of irreversibility
IQR	Inter-Quartile Range
mAD	Mild Alzheimer’s dementia
MCI	Mild cognitive impairment
PE	Permutation entropy
RF	Random forests
